# The social contingency of momentary subjective well-being

**DOI:** 10.1038/ncomms11825

**Published:** 2016-06-13

**Authors:** Robb B. Rutledge, Archy O. de Berker, Svenja Espenhahn, Peter Dayan, Raymond J. Dolan

**Affiliations:** 1 Max Planck University College London Centre for Computational Psychiatry and Ageing Research, London WC1B 5EH, UK; 2 Wellcome Trust Centre for Neuroimaging, University College London, London WC1N 3BG, UK; 3 Sobell Department of Motor Neuroscience and Movement Disorders, University College London, London WC1N 3BG, UK; 4 Gatsby Computational Neuroscience Unit, University College London, London W1T 4JG, UK

## Abstract

Although social comparison is a known determinant of overall life satisfaction, it is not clear how it affects moment-to-moment variation in subjective emotional state. Using a novel social decision task combined with computational modelling, we show that a participant's subjective emotional state reflects not only the impact of rewards they themselves receive, but also the rewards received by a social partner. Unequal outcomes, whether advantageous or disadvantageous, reduce average momentary happiness. Furthermore, the relative impacts of advantageous and disadvantageous inequality on momentary happiness at the individual level predict a subject's generosity in a separate dictator game. These findings demonstrate a powerful social influence upon subjective emotional state, where emotional reactivity to inequality is strongly predictive of altruism in an independent task domain.

## Abstract (web summary)

Comparing oneself to others is inherently human but exactly how social comparison affects one's emotional state is unclear. Here the authors demonstrate that unequal social outcomes decrease happiness and these emotional impacts are proportional to individual levels of generosity.

## Graphical abstract

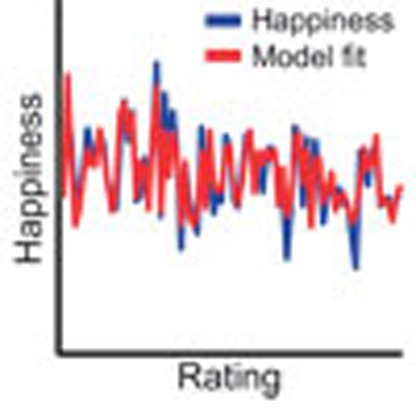

Subjective well-being is a key index of quality of life^1^,^2^, prompting policies aimed at increasing it^3^. However, maximizing wealth is not an effective way of maximizing well-being, as the coupling between the two is often relatively weak^4^,^5^,^6^,^7^ (although see ref. 8). Social comparison has been suggested as an important mediator of the relationship between wealth and well-being, with relative as opposed to absolute wealth exerting a substantial influence^9^,^10^,^11^.

Social comparison is also increasingly acknowledged as being relevant to economic behaviour. Aversion to advantageous and disadvantageous inequality is suggested to contribute to altruistic behaviour^12^,^13^. However, it is unknown whether variance in the emotional impact of inequality on well-being relates to the heterogeneity observed in altruistic behaviour^14^. Here, we address these issues at the level of the individual by examining the impact of social comparison on momentary subjective well-being. We show that, on average, the impact of inequality is to attenuate momentary happiness. Furthermore, the relative emotional impact of advantageous and disadvantageous inequality predicts altruistic behaviour at the level of individuals.

We previously quantified the relationship between rewards and momentary happiness using a probabilistic reward task, showing that momentary subjective well-being depends on the cumulative impact of recent expectations and the reinforcement prediction errors (RPEs) that arise from these expectations^15^. RPEs, the difference between experienced and expected outcomes, are thought to be encoded in the firing pattern of dopamine neurons^16^,^17^. In keeping with this, we observed that changes in subjective well-being were coupled to reward-related neural responses in the striatum, an area with rich dopaminergic innervation^15^. A dopaminergic mediation of this effect was also suggested by the observation that pharmacologically boosting dopamine increased well-being related to reward receipt^18^.

Here, we exploit our previously established computational approach to study how inequality, and putative emotional responses to inequality, impact on one important component of well-being. Our approach was motivated by prior observations that striatal neural responses can also reflect the rewards received by others^19^,^20^,^21^. This led us to predict that rewards received by another person would impact participants' momentary subjective well-being according to their individual social preferences. We also predicted that individual emotional reactivity to social outcomes might relate to heterogeneity in altruistic choice, something that has been difficult to explain using standard economic approaches^14^.

Our results show that a subject's subjective emotional state reflects rewards received by a social partner. Advantageous and disadvantageous inequality both reduce momentary happiness on average. Furthermore, we use computational modelling to show that the relative emotional impacts of advantageous and disadvantageous inequality predict a subject's generosity in a separate dictator game, suggesting that variability in the emotional impact of inequality on well-being can explain heterogeneity in altruistic behaviour.

## Results

### Measuring the impact of inequality on subjective well-being

**Figure 1:**
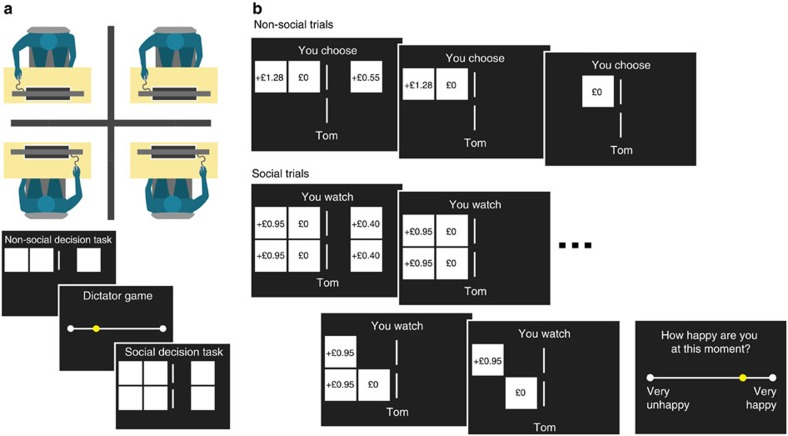
Experimental design. (**a**) Four participants were introduced to each other and seated in separate rooms to perform three tasks: a non-social decision task, a dictator game, and a social decision task. In the non-social decision task, subjects (*n*=47) chose between safe options and risky gambles with equal probabilities of two outcomes. In the dictator game, subjects decided how to split an endowment between themselves and another player. (**b**) The social decision task consisted of non-social and social trials. In non-social trials, choice outcomes (here £0) did not affect partner earnings. In social trials, subjects were told that they were observing choices made by their partner in the non-social decision task. When their partner chose to gamble, the subject received an equivalent but independent gamble. The subject's outcome was revealed first (here gaining £0.95), followed by the partner's outcome (here £0). After every 2–3 trials, subjects were asked to report their current level of happiness.

Our experimental design involved testing subjects in groups of four. Subjects (*n*=47) were first introduced to each other, then seated in separate rooms and asked to complete three different tasks (Fig. 1a). The first task was a non-social decision task^15^,^22^,^23^, in which subjects chose between safe and risky options. Subjects faced Gain trials (certain gain versus a gamble to gain a larger amount or zero), Mixed trials (zero versus a gamble to gain an amount or lose an amount) and Loss trials (certain loss versus a gamble to lose a larger amount or zero). Chosen gambles were resolved after a brief delay and the outcomes of all trials counted towards earnings. The second task was a standard economic task, the dictator game, in which a subject decided how to split an endowment (either £2 or £3, see the Methods for details) with one of the other players^24^. Importantly, these allocations were private and subjects were told that the monetary split would never be revealed to the other player. At the end of the experiment, subjects learned their total earnings for completing all tasks and were not told if, or how much, any other player had contributed to that total. The design feature whereby allocations were private is important because generosity might otherwise reflect primarily a reputational concern for what other players will think of them^25^. Generosity was estimated based on behaviour in the dictator game as the percentage of the endowment that subjects allocated to their social partner. This quantity varied between 0 and 50%, consistent with previous research^26^. The third and final task involved social and non-social decision trials, where subjects were again presented with safe and risky options (Fig. 1b). In the non-social trials, subjects made choices as in the first task. In the social trials, subjects were shown two sets of identical safe and risky options and informed that one set was allocated to them, and the other set corresponded to a trial the partner had previously experienced in the non-social decision task. Subjects were informed that on these trials they could not make a decision for themselves, but observed, and were subject to the outcomes of the choices that the partner had previously made. In reality, choices on social trials were generated using a standard decision model based on prospect theory, using parameters for a typical subject (see the Methods for details). This procedure ensured that all participants had a similar experience in social trials.

When the partner chose the safe option, both players received the same outcome; when the partner chose the risky option, both players received the gamble. The critical manipulation centred on the independence of the two gambles for the subject and the partner. This meant that for any single gamble chosen by the partner, the outcomes experienced by the subject and their partner could be identical or different (Fig. 1b), providing the potential for inequality. In all trials, the outcomes for the subject counted towards overall earnings. To investigate the relationship between subjective emotional state and the outcomes of choices, including choices made by others, we used experience sampling^15^,^27^,^28^, repeatedly asking subjects, ‘How happy are you at this moment?' after every 2–3 trials. Subjects were tested using two slightly different procedures (see the Methods for details) with an identical trial structure, and were informed of total earnings only after completion of all tasks. Although happiness due to inequality could potentially be measured without any choice on the subject's part, not being able to make any choices would reduce engagement, and the results of our previous studies show that outcomes resulting from a subject's choices substantially impact happiness^15^,^18^. Thus, we interleaved social and non-social trials, and outcomes for the two types of trials were independent, allowing us to dissociate these influences.

We first examined the determinants of subjective well-being, and found, consistent with our previous research^15^, that subjects reported greater average happiness at the subsequent rating after winning compared with losing gambles in both social and non-social trials (Wilcoxon signed-rank test, *n*=47, both *Z*>4, *P*<0.001). In social trials, we tested whether there was an impact of partner outcomes on well-being by *z*-scoring ratings for each subject and computing average happiness at the subsequent rating across the following four contexts: both participants win, both participants lose, subject wins and partner loses and subject loses and partner wins. The last two conditions are associated with advantageous (subject has more) and disadvantageous (subject has less) inequality, respectively. These two contexts are ones that might engender the social emotions of guilt and envy, respectively, emotions that have parallels with terms in models of altruistic behaviour^12^.

### Inequality reduces subjective well-being

**Figure 2:**
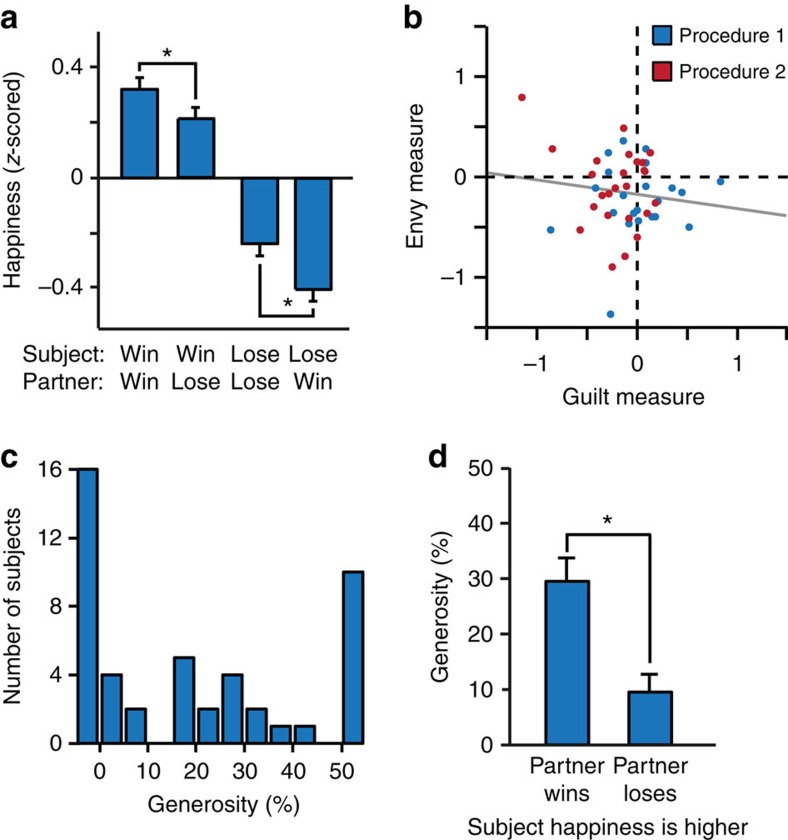
Descriptive analysis. (**a**) Subjects (*n*=47) reported being happier at the subsequent rating after winning compared with losing gambles, and happiness ratings were lower on average when the partner received a different outcome, regardless of whether that outcome was better or worse. (**b**) The amount that happiness was affected by advantageous inequality (guilt is when subject wins and partner loses minus subject wins and partner wins) and disadvantageous inequality (envy is when subject loses partner wins minus subject loses and partner loses) was uncorrelated across subjects (Spearman's *ρ*=−0.04, *P*=0.78). (**c**) Subjects completed a dictator game in which they could anonymously give a fraction of an endowment to their partner; the bar farthest to the right indicates that 10 subjects gave half of the endowment and the bar farthest to the left indicates that 16 subjects gave nothing. (**d**) Subjects were more generous in the dictator game if their happiness in the separate social decision task was higher when the partner won than lost gambles. The difference between guilt and envy measures was correlated with generosity in the dictator game (Spearman's *ρ*=−0.48, *P*<0.001). Subjects who were happier after the partner lost than won gambles included only 2 of 10 subjects who gave half but 12 of 16 subjects who gave nothing. Error bars, s.e.m. *P<0.05.

We found that, regardless of whether subjects themselves won or lost, average subjective well-being was attenuated for unequal compared with equal outcomes. Well-being was reduced both when subjects were better off (*Z*=−2.2, *P*=0.028) and when they were worse off (*Z*=−2.8, *P*=0.005) than the other person (Fig. 2a). We tested the possibility that the sensitivity of subjective well-being to advantageous and disadvantageous inequality (referred to here as guilt and envy) is equivalent, as might be expected if it reflected a unified concept of inequality aversion. However, we found no correlation between the change in well-being when subjects were better compared with worse off than their partner (Spearman's *ρ*=−0.04, *P*=0.78; Fig. 2b), suggesting independent variation in the degrees of guilt and envy, a result inconsistent with a unified concept of inequality aversion.

### Emotional impacts of inequality relate to generosity

We next determined whether a social influence on well-being was related to generosity in the entirely separate dictator game. For each subject, we computed the difference in happiness between when the partner loses and when the partner wins, equivalent to taking the difference between guilt and envy measures. Against a backdrop of a 20% average allocation in the dictator game (Fig. 2c), consistent with previous studies^26^,^29^, subjects who were more happy on average when the partner wins than when they lose were also more generous in the dictator game, compared with subjects who were less happy when the partner wins than when they lose (Wilcoxon rank-sum test, *Z*=3.4, *P*<0.001; Fig. 2d). Strikingly, the first group of subjects gave three times as much of their endowment on average than the second group (30% versus 10%). Generosity in the dictator game was highly correlated with the difference between guilt and envy measures derived from happiness ratings (Spearman's *ρ*=−0.48, *P*<0.001). Although generosity might be thought to depend on the guilt of receiving an unexpected endowment, we found that generosity was not significantly correlated with guilt measures alone (Spearman's *ρ*=−0.18, *P*=0.22) but was correlated with envy measures (Spearman's *ρ*=0.30, *P*=0.042) such that subjects exhibiting greater envy were less generous, a pattern of results inconsistent with any plausible demand characteristics of the experimental design.

### Modelling the impact of inequality aversion on well-being

Our next goal was to apply our previously established methodology for measuring determinants of momentary subjective well-being to quantify individual dispositions in the social domain that impact emotional reactivity. Our starting point was our pre-existing non-social happiness model^15^, in which chosen certain rewards (CR), the expected value (EV) of chosen gambles and RPEs resulting from those expectations, all exert separate influences that decay exponentially with time:


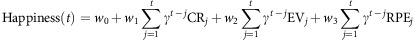


where *t* is trial number, *w*_0_ is a constant term, other weights *w* capture the influence of different event types, 0≤*γ*≤1 is a forgetting factor that makes events in more recent trials more influential than those in earlier trials, CR_*j*_ is the certain reward if chosen instead of a gamble on trial *j*, EV_*j*_ is the average reward for the gamble if chosen on trial *j* and RPE_*j*_ is the RPE on trial *j* contingent on choice of the gamble. Terms for unchosen options were set to zero. We *z*-scored happiness ratings to prevent subjects with greater variability in their ratings from having a disproportionate effect on results. The constant term is omitted when ratings are *z*-scored. We fitted parameters to the happiness ratings of individual subjects in the social decision task and found, as expected, that CR, EV and RPE weights were on average positive (all *Z*>4, *P*<0.001). The forgetting factor *γ* was 0.67±0.25 (mean±s.d.) indicating that ratings on average depended on the cumulative impact of five to ten past events. Despite having no way to account for any social effect, this model explained momentary happiness well, with *r*^2^=0.39±0.19 (mean±s.d.), comparable to fits for a non-social task in a previous study^15^.

**Figure 3:**
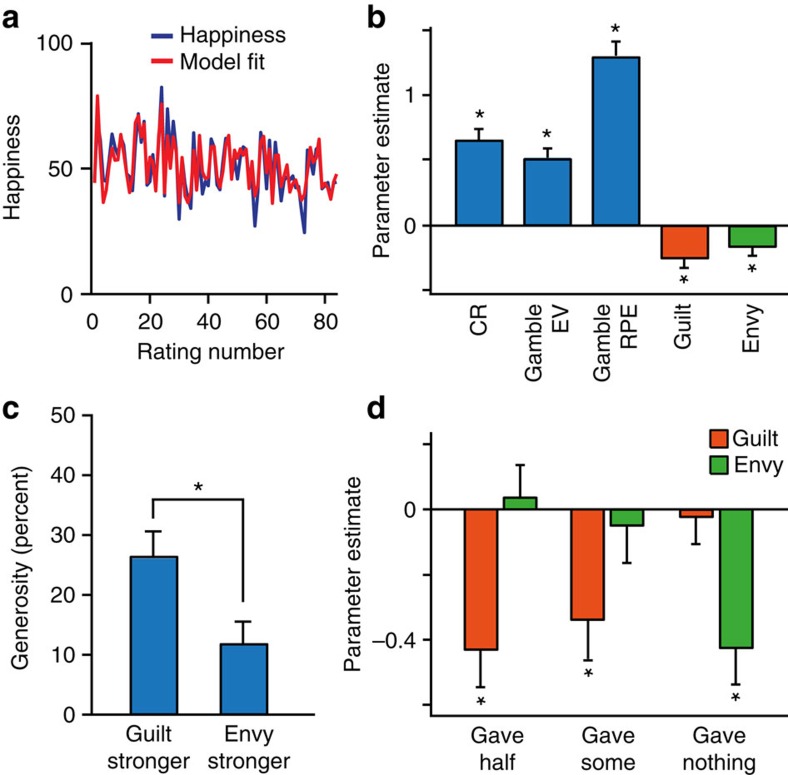
Model-based analysis. (**a**) Happiness ratings of an example subject over the course of the experiment plotted with the predictions of the guilt–envy model. (**b**) Happiness was affected by model parameters (*n*=47) related to the subject's rewards. Two additional model parameters related to inequality aversion were both negative, indicating that both advantageous inequality (guilt parameter) and disadvantageous inequality (envy parameter) negatively impact happiness on average. (**c**) Subjects with stronger (more negative) guilt parameters were more generous in the separate dictator game than subjects with stronger (more negative) envy parameters. The difference between guilt and envy parameters was correlated with generosity in the dictator game (Spearman's *ρ*=−0.48, *P*<0.001). (**d**) Guilt and envy parameters estimated by the model for subjects with different levels of generosity in the dictator game. Subjects who gave nothing had significant envy parameters. Subjects who gave something had significant guilt parameters. Error bars, s.e.m. *P<0.05.

We next expanded the model by including additional terms to account for influences related to advantageous and disadvantageous inequality^12^. These influences might be considered as related to guilt and envy, respectively:


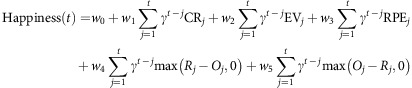


where *w*_4_ relates to advantageous inequality (guilt) when the reward received by the subject R_*j*_ exceeds the reward received by the other player *O*_j_, and *w*_5_ relates to disadvantageous inequality (envy) when *O*_*j*_ exceeds *R*_*j*_. This guilt-envy model explained momentary happiness better than its non-social variant with *r*^2^=0.44±0.18 (mean±s.d.; Fig. 3a). This model was preferred to the simpler non-social model in a Bayesian model comparison, which penalizes for the number of parameters^30^,^31^ (see Table 1 for details), demonstrating that social comparison significantly impacts subjective well-being in our task. Model parameters for guilt and envy were negative on average (both *Z*<−2, *P*<0.05; Fig. 3b), consistent with both advantageous and disadvantageous inequality reducing momentary happiness.

### Envy and guilt parameters predict generosity

When we tested how model parameters related to individual social preferences, we found subjects with stronger (more negative) guilt parameters were more generous in the dictator game than subjects with stronger (more negative) envy parameters (*Z*=2.8, *P*=0.006; Fig. 3c). Consistent with the descriptive analysis, the difference between guilt and envy parameters estimated from happiness ratings was highly correlated with generosity in the dictator game (Spearman's *ρ*=−0.48, *P*<0.001). Guilt but not envy parameters were significantly negative for subjects that altruistically gave either half or some of the endowment (guilt, both *Z*<−2.3, *P*<0.05; envy, both |*Z*|<0.5, *P*>0.5; Fig. 3d), whereas the opposite was true for those subjects that gave nothing (guilt, *Z*=−0.2, *P*=0.88; envy, *Z*=−2.9, *P*=0.003).

One concern is whether demand characteristics might contribute to any of our results. Some subjects might have noticed that inequality was one feature of the experiment and hypothesized that well-being should reflect a unitary concept of inequality (‘inequality is bad'). To test whether this possibility could explain our results, we fitted an additional model with a term for the magnitude of the difference in rewards between players. The inequality parameter in this simple-inequality model was significantly negative on average (*Z*=−5.13, *P*<0.001), capturing lower well-being with greater inequality (see the Methods for details). However, this inequality parameter was uncorrelated with generosity in the dictator game (Spearman's *ρ*=−0.036, *P*=0.81), which might theoretically have responded to the same demand characteristic, and the guilt–envy model outperformed the simple-inequality model according to Bayesian model comparison (Table 1).

## Discussion

Our results provide striking quantitative confirmation that an individual's subjective reports of momentary well-being in a social context reflect not only how well things are going relative to expectations, but also how things are going relative to other people, even when outcomes for others are both independent from, and irrelevant to, the subjects' own earnings. By quantifying social preferences based on emotional reactivity to inequality separately from economic choice, we avoid strategic considerations, a potential confound in using economic games to understand the role of inequality aversion in behaviour^32^. Furthermore, by using the continuously varying subjective state as an output measure, we avoid forcing subjects to explicitly admit to emotions that might be perceived to have negative social connotations, such as envy.

Increasing inequality in many countries^33^,^34^ lends urgency to the need to understand the impact of this disparity, both at individual and societal levels^35^. Our demonstration that inequality aversion reduces momentary well-being aligns with wider observations of inequality's negative impact on societal well-being^36^. Culturally entrained aversion to inequality such as ‘Janteloven' observed in Scandinavia^37^ may therefore play an important role in shaping well-being in those countries, which rank highly in international well-being surveys. The fact that individual differences in well-being measures were predictive of social preferences suggests these parameters reflect values that are at least part of a stable variation in generosity between individuals^38^, variation that has been difficult to explain using economic approaches alone^14^. Our findings also highlight an important issue for future research: our subjects experienced inequality in social trials where they were unable to influence their partner's decision. Understanding how instrumental control impacts well-being could shed additional light on the mediating role of agency in the emotional impacts of societal inequality.

We adopted a quantitative model that opens up new avenues to investigate the relationship between subjective well-being and behaviour. Although numerous studies have linked experienced and anticipated emotions during choice to subsequent behaviour (reviewed in ref. 39), it has remained unclear whether choices accurately anticipate the emotional impact of outcomes on subjective well-being, with some arguing that these quantities are distinct^40^. However, recent economic research suggests that quantitative links can be forged between hypothetical choices and hypothetical consequences for well-being^41^. Here, we demonstrate a precise link between subjective well-being following actual rewards and incentivized economic altruistic choice.

There is considerable debate as to the underlying basis for altruistic behaviour. Although Fehr and Schmidt posit that guilt and envy relate to altruistic behaviour^12^, it has never been tested whether emotional responses to advantageous and disadvantageous inequality explain heterogeneity in generosity. We found that the relative emotional impact of guilt and envy is predictive of generosity, lending support to the Fehr–Schmidt model. However, our results are inconsistent with two assumptions of this model, specifically that weights for guilt and envy are correlated and that the weight for envy is greater than the weight for guilt. We found that the emotional impacts of advantageous and disadvantageous inequality are uncorrelated such that people who experience more guilt do not necessarily experience more envy. Furthermore, in our model fits, we found that guilt parameters were greater than envy parameters in the majority of our subjects, in sharp contrast to an assumption of the Fehr–Schmidt model. This result is relevant to altruistic behaviour in that participants who had larger guilt than envy parameters were on average more generous than individuals for whom the converse was true (Fig. 3c). Our results therefore recommend alternate social-welfare preference models that relax the assumptions of the Fehr–Schmidt model^42^,^43^.

Our emotional dissection of inequality aversion also addresses an important critique that emerges from the constraints of dictator games, namely that any action other than keeping all of the money looks like inequality aversion^42^. Inattentive subjects could inadvertently appear altruistic. Our results show that much of the variance in generosity cannot be explained by this concern, because noisy happiness ratings could not be misconstrued as evidence for inequality aversion. Our finding of a link between emotional measures and generosity provide a new perspective on the value of dictator games as assays of social preferences.

Recent work on the ontogeny of fairness across cultures finds that an aversion to disadvantageous inequality arises early in the development, but that an aversion to advantageous inequality arises later, and appears possibly for strategic reasons^13^. Similarly, dual-process models suggest that prosocial behaviour might result from an interaction between intuitive/emotional and deliberative/non-emotional processes^44^,^45^. However, we find that emotional processes alone are sufficient to explain heterogeneity in generosity, without invoking strategic concerns or conflict between emotional and non-emotional processes. This result also argues against the need to appeal to any understanding of the emotional state of another person, as in popular empathy models^46^, at the time that altruistic decisions are made.

Demand characteristics can be a concern in the study of both well-being and altruism. However, there are several reasons why a demand-driven explanation of our results is unlikely. First, the task was designed such that the rewards of others are irrelevant to earnings, reducing the chance that subjects will realize that their ratings reveal a socially undesirable emotion-like envy. Evidence that this was successful is that most (∼90%) of the variance in ratings accounted for by the model arose from non-social influences, influences known to be the same in paid lab subjects and unpaid anonymous subjects^15^, inconsistent with significant demand effects in the non-social influences on well-being. Second, the average forgetting factor is such that ratings reflected the cumulative influence of five to ten past events. However, ratings were made too quickly (on average in 1.7 s in the social decision task) to allow the sort of deliberate calculation that demand effects over such a timescale might require. Third, we fitted a simple-inequality model that captures the unitary concept of inequality (‘inequality is bad') that is most likely to be consistent with perceived experimenter demands. This model did not explain variation in generosity, and did not explain the data as well as the guilt–envy model. A final argument against any explanation in terms of demand characteristics is that we observe a strong effect of envy on well-being in subjects who also appear sufficiently immune to experimenter demands as to give nothing in the dictator game (Fig. 3d).

Our computational approach might be fruitfully employed to meet a variety of challenges. The most immediate application is in testing hypotheses regarding the role of emotions in prosocial behaviour across economic games, including social and moral emotions that might relate to behaviours such as trust and punishment. Furthermore, computational models such as ours can provide precise subject-specific predictions for interrogating the neural circuits that support prosocial behaviour while also generating predictors related to negative emotions such as guilt and envy that can be difficult to elicit explicitly in experimental settings. Understanding individual differences in the determinants of well-being may also yield insight into interactions between people of different socioeconomic status, which may have economic implications. Finally, individual phenotyping based on emotional dynamics could provide a powerful tool to dissect social pathologies, such as borderline personality disorder.

## Methods

### Participants

Forty-seven healthy subjects took part in the experiment (age range 18–39 years, 22 males), using two slightly different procedures (*n*=22 and *n*=25). Same-gendered subjects who did not know each other were tested in groups of four and confederates were used when one of the scheduled subjects was absent. The experimenter asked subjects to introduce themselves to the other members of the group before seating them in four separate rooms. All subjects gave informed consent and the Research Ethics Committee of University College London approved all studies.

### Experimental procedure

Stimuli were presented using Cogent 2000 (Wellcome Trust Centre for Neuroimaging) in MATLAB (MathWorks, Inc.). First, subjects completed a non-social decision task with 140 trials. Subjects completed instructions and a practice session before the task. On each trial, subjects made a choice between a safe and a risky option, which was resolved after a 2.5-s delay period^15^,^18^,^22^. Subjects faced Gain trials (certain gain versus a gamble to gain a larger amount or zero), Mixed trials (zero versus a gamble to gain an amount or lose an amount) and Loss trials (certain loss versus a gamble to lose a larger amount or zero). Options presented in the task were similar to those used in a previous study^15^. Subjects had 5 s to make their decision and otherwise received the worst outcome from the gamble. The position of safe and risky options was left–right reversed every ten trials.

To familiarize subjects with answering questions about their subjective emotional state, during the non-social decision task, we used the same key measure obtained in the social decision task, asking subjects the question ‘How happy are you at this moment?' at the start of the task and after every ten trials. The left side of the line was marked ‘very unhappy' and the right side of the line was marked ‘very happy'. Subjects moved a cursor to indicate their current subjective state. The cursor always started at the midpoint. Subjects had a 5-s time limit to make their responses and the current cursor position was entered as the response if they did not respond within the time limit. The average decision time was 1.4 s and the average rating time was 2.0 s in the non-social decision task. Total task earnings were not revealed to subjects during the experiment; subjects were told that the computer would track all of their earnings and they would be told the combined total for all tasks and receive those earnings at the end of the experiment. Although this task only included 15 happiness ratings, we fitted our pre-existing non-social happiness model to *z*-scored happiness ratings and found, as expected, that CR, EV and RPE weights were on average positive (means: CR=0.83, EV=0.62, RPE=1.15; all *Z*>4, *P*<0.001). The forgetting factor was 0.65±0.31 (mean±s.d.).

Second, subjects completed a dictator game in which they were endowed with an amount of money and tasked with splitting the money between themselves and a named partner. They were told that the split was anonymous and would be added to the partner's total earnings without the partner's knowledge. Subjects had no time limit to make their decisions in the dictator game. The social decision task that immediately followed was with the same named partner. In procedure 1 (*n*=22), subjects were paired with only one partner, completing a single dictator game with an endowment of £3. In procedure 2 (*n*=25), subjects were paired sequentially with two partners, completing a dictator game with an endowment of £2 before a social decision task with each partner. We employed this procedure to determine if there was any variability in generosity within subjects that could be exploited to examine differences in emotional reactivity to outcomes received by the two partners. No happiness ratings were collected during the dictator game.

Generosity in the dictator game was almost identical on average between procedure 1 and procedure 2 (20% versus 19%; *Z*=0.09, *P*=0.93). However, generosity in the dictator game in procedure 2 was highly correlated between the two partners (Spearman's *ρ*=0.88, *P*<0.001), suggesting that generosity in this task is stable, at least with unfamiliar partners. To further ascertain if subjects had any preference for one of the partners that might impact generosity, at the end of the experiment we asked subjects which unfamiliar partner they would prefer to have a conversation with. There was no difference in generosity towards preferred and non-preferred partners (*Z*=−1.09, *P*=0.27). Owing to the high degree of similarity in generosity across repeated dictator games in procedure 2, we combined data from the two partners and took the mean of generosity in the two dictator games.

Third, subjects completed a social decision task in which on each trial they were again presented with a safe and a risky option. The task consisted of non-social and social trials, with the order pseudo-randomized to ensure that there were never more than two non-social trials or four social trials in a row. In non-social trials, subjects chose as in the non-social decision task and the outcome of decisions was added to their earnings. Subjects had 5 s to make their decision and otherwise received the worst outcome from the gamble. In social trials, they were told that they were observing the choice made by the social partner when that partner earlier completed the non-social decision task. If the partner chose the safe option, then that outcome was added to their earnings. If the partner chose the gamble, then independent gambles were resolved for the subject and the partner. The subject's outcome was resolved first after a 2.5-s delay period. The partner's outcome was resolved after an additional 2.5 s delay period. Subjects were asked after every two or three trials ‘How happy are you at this moment?' providing us the opportunity to examine the effect of inequality on subjective emotional state. All outcomes received by the partner were purportedly obtained earlier in the experiment in a non-social context and there was no way for subjects to influence those choices in any way.

Decisions in social trials were not in fact made by the partner but were made by the computer in a manner appropriate for an agent with typical economic preferences. This agent made choices based on the parametric prospect theory model with typical loss aversion (*λ*=1.35) and typical risk aversion in gains and risk seeking in losses (*ρ*=0.9). These parameters are similar to average parameter values obtained in a non-social experiment with a similar design^22^.

In procedure 1, the social decision task involved 210 trials (70 non-social trials and 140 social trials) including 85 happiness ratings. In procedure 2, each social decision task involved 150 trials (50 non-social trials and 100 social trials) including 61 happiness ratings. The percentage of trials in which subjects chose to gamble was similar in non-social and social decision tasks (median of 54% in both tasks) and similar to the percentage of trials in which the computer partner gambled (median of 55%). In the social decision task, the average decision time in the non-social trials was 1.6 s and the average rating time was 1.7 s. Subjects completed on average 99% of non-social trials and entered ratings on average in 96% of trials within the time limit.

### Descriptive and model-based analyses

Happiness ratings were *z*-scored for all analyses so that subjects with greater variability in their ratings did not disproportionately contribute to results. Owing to the non-normality of decisions in the dictator game, with 26 subjects giving either half or nothing (Fig. 2b), we used non-parametric statistical tests including two-tailed Wilcoxon signed-rank tests, Wilcoxon rank-sum tests and Spearman's rank correlations. Unless otherwise stated, all statistical tests included all 47 subjects.

We modelled momentary happiness using models that assume an exponential decay in the influence of previous events^15^,^18^. Models were fit to ratings in individual subjects using nonlinear least squares using the optimization toolbox in MATLAB (Mathworks, Inc.). *Z*-scoring produces ratings with a mean value of 0, eliminating the need for a constant term in the model. The non-social model accounted for at least 10% of the variance in ratings for 45 of 47 subjects. The simple-inequality model included a parameter for the magnitude of the difference in rewards between the two players. The guilt-envy model included parameters for the magnitude of advantageous inequality (guilt) and the magnitude of disadvantageous inequality (envy). These parameters will be negative if either type of inequality reduces well-being. We used Bayesian model comparison to compare models^30^,^31^. We computed Bayesian Information Criterion (BIC) measures for each individual model fit and summed across subjects. BIC is a measure that quantifies the deviation of the model's predictions from the data. A lower BIC value is therefore preferable. However, BIC also penalizes for the number of parameters, allowing the direct comparison of models with different numbers of parameters. Because the relative BIC value is important, and not the absolute BIC value, we also computed the BIC values relative to the winning model (Table 1).

### Data and code availability

All data and code necessary to reproduce the results reported are available on request to the corresponding author.

## Additional information

**How to cite this article:** Rutledge, R. B. *et al*. The social contingency of momentary subjective well-being. *Nat. Commun.* 7:11825 doi: 10.1038/ncomms11825 (2016).

## Figures and Tables

**Table 1:** Bayesian model comparison analysis.

Model	Parameters per subject	Mean *r*^2^	Median *r*^2^	Model BIC	Relative BIC
Non-social	4	0.39	0.37	−1723	+72
Simple-inequality	5	0.41	0.40	−1704	+91
Guilt–envy	6	0.44	0.47	−1795	0

BIC, Bayesian Information Criterion.

BIC values are summed across the 47 subjects. Model fits were performed with *z*-scored happiness ratings. All three models contained separate terms for CRs, gamble EVs and gamble RPEs, with influences that decay exponentially. The simple-inequality model included an additional parameter for the magnitude of the difference in outcomes between the two players. The guilt–envy model included additional parameters for advantageous and disadvantageous inequality. The guilt–envy model had the lowest BIC and therefore is preferred by Bayesian model comparison.
